# Proceedings of the De Witt County Medical Society

**Published:** 1860-12

**Authors:** Christopher Goodbrake

**Affiliations:** Secretary


### PROCEEDINGS OF THE DE WITT COUNTY MEDICAL SOCIETY.

Reported by CHRISTOPHER GOODBRAKE, M.D., Secretary.

The Society met in Semi-Annual Session, at the Office of Drs. Wright & Davis, in Wappella, on Tuesday, the 2nd day of October, 1860. Dr. John Wright in the Chair.

The Minutes of the previous Meeting were read and approved.

Dr. Thomas W. Davis, who had previously passed the Board of Censors, was, on motion of Dr. Goodbrake, elected a member of the Society.

Dr. Wright reported a case of wounded knee joint. The patient, a man about 45 years of age, had received a small wound with a knife, such as is used for cutting up corn, immediately below the patella, on the inner side of the knee. The joint swelled very rapidly and became extremely painful. It was heated with warm fomentations to the knee, and quinine, opium and calomel administered internally. The case was progressing very favorably.

Dr. Davis reported three cases of typhoid fever that came under his notice, which presented some singular features. The patients, in the three cases, had all the usual symptoms of this disease; such as head-ache, lassitude, want of appetite, tongue coated with a whitish fur very narrowly edged with red, bowels more or less tender with a disposition to diarrhœa; pulse from 95 to 110 per minute. These symptoms lasted from two to three weeks; the patients all this time being able to go about the house; and what was singular they became as much emaciated during the period of their sickness as others whom the doctor attended at the same time, and who became very low, so much so, as not to be able to turn themselves in the bed. The doctor's treatment consisted in tonics, animal broths, with opium and nitrate of silver—$\frac{1}{4}$ grain of each every six hours—when considered necessary.

Dr. Goodbrake reported two cases of puerperal convulsions; both were primapara cases. The first was that of a woman about 30 years of age; the convulsions set in about the time the os tincæ began to dilate. She was bled freely; chloroform was administered to control, as far as possible, the convulsions. She was delivered, by the aid of the forceps, of a living child, nine hours after the accession of the first convulsion. The woman died 24 hours after the delivery, in a comatose condition.

The second case was that of a Mrs. J——, aged 17 years, of a small stature, short neck, florid countenance, sanguine temperment. This patient was *not bled*, but chloroform was administered to control the convulsions; unguentum belladonna was applied very freely to the mouth of the womb; she was delivered by the aid of instruments, six hours from the time she had her first convulsion, of a dead child. The patient made a good recovery. The doctor gave it as his experience that a large percentage of puerperal convulsions occurred in primapara cases; also that they took place about the time when the os tincæ commenced dilating, which was usually found rigid and unyielding; and was of the opinion, that in such cases the early and free application of the belladonna ointment might prevent these frightful convulsions. He said that he believed he was warranted in saying, that in several cases where this horrible disease was threatening, he averted it by this treatment.

Cholera infantum, the regular subject for discussion, was then taken up; when most of the gentlemen present expressed their views on the pathology and treatment of the disease.

Drs. W. W. Adams and T. K. Edmiston, were appointed Essayists.

On motion, the thanks of the Society were tendered to Drs. Wright and Davis, and their ladies, for the sumptuous dinner served up for the members at this session.

Typhoid Fever was chosen as the regular subject for discussion at the next meeting.

The Society then adjourned to meet in Quarterly Session, at Marion, on the first Tuesday of January, 1861.

